# Arsenic Makes Its Mark: Using Biomarkers to Track Noncancer Respiratory Effects

**Published:** 2008-02

**Authors:** M. Nathaniel Mead

Exposure to toxic levels of arsenic is a serious problem in many Asian countries, notably Bangladesh and India, where exposure to inorganic arsenic through naturally contaminated groundwater is widespread and often excessive. Scientists have long recognized the lung to be a major site of action of ingested arsenic, and most of the focus has been on risks associated with lung cancer. Nonetheless, habitual ingestion of high-arsenic drinking water also promotes many noncancer lung effects, and researchers are investigating the potential utility of biomarkers to identify such effects **[*EHP* 116:190–195; Parvez et al.]**.

One of the challenges of pursuing connections between arsenic and respiratory illness is that assessments of respiratory symptoms may be prejudiced by interviewer bias if study participants show visible skin lesions, a hallmark sign of chronic arsenic poisoning. To overcome this obstacle, researchers have begun exploring the use of biomarkers for chronic respiratory disease such as serum levels of Clara cell protein CC16. This serum marker runs low in individuals with compromised lung conditions induced by chronic environmental exposures such as cigarette smoking or ozone.

In the current study, investigators sought to determine the relationships of serum CC16 with well-water arsenic, total urinary arsenic, and urinary arsenic methylation indices in a population of 241 nonsmoking individuals exposed to arsenic-laden drinking water in Araihazar, Bangladesh. The mean arsenic concentration in drinking water was 134 μg/L, and individuals with skin lesions consumed significantly more arsenic than those without (159 versus 105 μg/L, respectively).

In individuals with skin lesions (but not those without such lesions), there was a significant inverse association of CC16 with urinary arsenic as well as a marginally significant inverse association of CC16 with the cumulative arsenic exposure index, which estimates long-term exposure. The researchers speculate that individuals with skin lesions either are exposed to higher levels of arsenic or have a unique susceptibility to the respiratory effects of arsenic exposure for reasons unknown.

The analysis also revealed positive associations of CC16 levels with the secondary arsenic methylation index (an indicator of arsenic methylation capability), particularly among individuals without skin lesions. This suggests that individuals with better methylation capacity may be less susceptible to the adverse respiratory effects of arsenic. Moreover, the inverse association of CC16 with the percentage of mono-methylated arsenic (a commonly measured arsenic metabolite) in urine indicates that individuals with incomplete methylation may be more vulnerable to arsenic-induced respiratory problems.

This cross-sectional investigation, the first to use biomarkers of arsenic exposure and lung injury in this way, is an important step toward further improving our understanding of arsenic-related non-malignant respiratory illness. Serum CC16 shows promise as a biomarker for assessing early respiratory damage induced by arsenic, especially among individuals with skin lesions associated with the consumption of arsenic-contaminated drinking water.

## Figures and Tables

**Figure:**
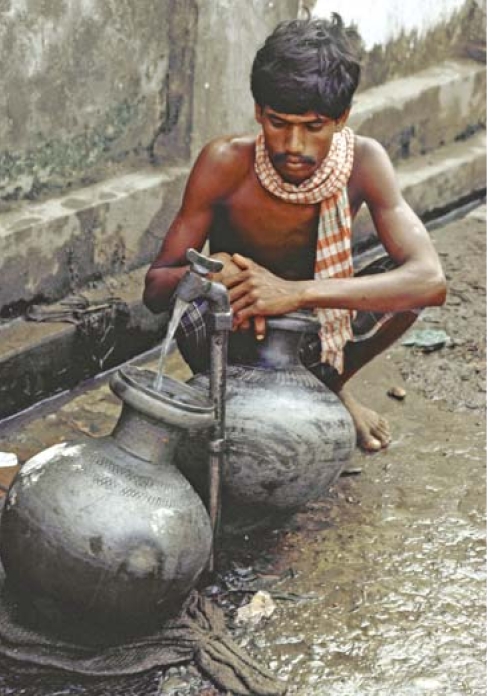
As it happens . . . Lung cancer isn’t the only respiratory effect linked with drinking arsenic-contaminated water; noncancer effects also may occur.

